# Self-assembled cell-scale containers made from DNA origami membranes

**DOI:** 10.1038/s41563-025-02418-0

**Published:** 2025-11-28

**Authors:** Christoph Karfusehr, Markus Eder, Hao Yuan Yang, Brice Beinsteiner, Marion Jasnin, Friedrich C. Simmel

**Affiliations:** 1 Department of Bioscience, TUM School of Natural Sciences, Technical University Munich, Garching, Germany; 2 Max Planck School Matter to Life, Heidelberg, Germany; 3 Helmholtz Pioneer Campus, Helmholtz Munich, Neuherberg, Germany; 4 Department of Chemistry, Technical University Munich, Garching, Germany

**Keywords:** DNA nanostructures, Membrane structure and assembly

## Abstract

Biological compartmentalization creates and controls localized environments to ensure that chemical processes are efficient, thus enabling life’s complexity and functionality. Biological systems use crystalline protein cages for nanoscale compartments, whereas larger, dynamic structures, such as vesicles and cell membranes, are formed from lipid bilayers. Although membrane-based approaches have prevailed in bottom-up synthetic biology, DNA and protein nanotechnology has focused on designing rigid cage assemblies. Here we report on the self-assembly of radially symmetric DNA origami subunits that are inspired by the structure and interactions of lipids. The formed DNA origami monolayer membranes can be readily programmed to form vesicles or hollow tubes with diameters ranging from 100 nm to over 1 μm. These DNA origami membranes represent an approach for compartmentalization that opens possibilities in bottom-up biology and cell-scale soft robotics.

## Abstract (web summary)

Through the programmable self-assembly of lipid-inspired radially symmetric DNA, porous molecular membranes and cell-sized compartments are formed with applications in bottom-up biology and soft robotics.

## Main

Compartmentalization is a hallmark of living systems and is used across several length scales in biology. By creating and controlling local chemical environments, spatially separated functional units are defined, thus enabling the hierarchical organization of living matter into higher-order structures. Ultimately, such organization is responsible for the complexity and diversity of life observed on Earth. Compartmentalization will also probably play a crucial role in the development of artificial bio-inspired systems at larger scales.

In biology, encapsulation is achieved within hollow containers made from proteins, lipids or their mixtures. When virus capsids or bacterial organelles are used for encapsulation, proteins assemble into rigid cage-like assemblies that often display quasi-icosahedral symmetry. On the scale of hundreds of nanometres and above, biology tends to use less well ordered and more dynamic assemblies, in particular lipid bilayer membranes and membraneless organelles based on liquid–liquid phase separation^1^. Lipid bilayer membranes are formed from monomeric subunits with flexible hydrocarbon chains^2^ that are held together by the hydrophobic effect and weak monomer–monomer interactions. In contrast to viral capsids^3^, the generation of global membrane curvature in these assemblies does not depend on the placement of curvature-inducing disclinations but emerges from the dynamic, local interactions of cone-shaped lipids^2^.

A wide range of efforts have recently focused on designing and using synthetically generated containers for compartmentalization. Among others^4–8^, this has involved the engineering of natural^9^ or de novo-designed protein cage formers^10–13^ and the creation of capsid-mimicking DNA cages that were self-assembled from small numbers of rigid DNA origami monomers^14,15^. By mimicking the assembly of membrane vesicles from phospholipids, other types of amphiphilic molecules have been used to realize peptidosomes^16,17^, polymersomes^18,19^ or DNAsomes^20^. Such analogues share the polymorphicity and polydispersity associated with lipid membranes, which are used in bottom-up synthetic biology^21^.

Although ordered cage-like assemblies enable the display of regular molecular patterns at their surfaces^22^ and often result in monodisperse assembly morphologies^23^, they do not easily permit alterations in geometry or functional characteristics. The creation of larger, capsid-like assemblies that are based on unique interactions is challenged by the higher number of distinct monomers involved and requires optimized assembly pathways^24^ and extended assembly times^25^. By comparison, membranes and phase-separated compartments form more readily and quickly but do not provide the addressable spatial order of the cage-like assemblies.

**Fig. 1:**
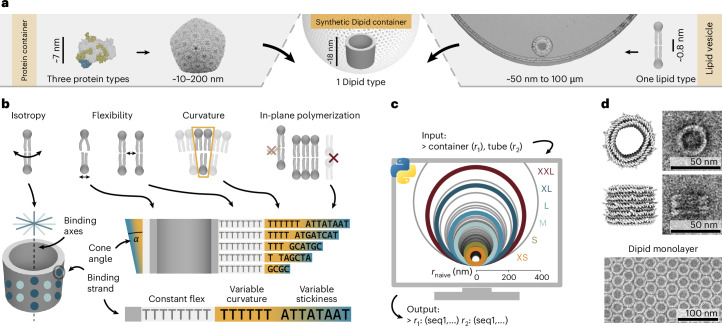
Design of lipid-inspired DNA origami monomers (‘Dipids’). **a**, Comparison of an *α*-carboxysome, formed from three distinct types of proteins^47,48^, small and large membrane vesicles formed from phospholipid bilayers, and an idealized Dipid container assembled from Dipid monomers. **b**, Mimicking lipid assembly with a DNA origami barrel. Isotropic interactions are approximated by 30 binding strands (dark and light blue spots) distributed on two sixfold axes. Monomer compliance is achieved by deliberately using barrels with thin walls (grey). Flexible monomer–monomer connections are facilitated by oligo-dT domains (light grey). The asymmetric extension of such ‘flex’ domains results in cone-shaped monomers, which assemble into structures with a global curvature controllable through the cone angle (orange to blue). The growth of monolayers from oriented monomers is enforced using a set of weak, self-complementary sticky domains (blue). **c**, The Dipid design script uses a worm-like chain polymer model to compute sets of curvature-domain extensions, which encode the cone angles of the Dipid and the expected assembly radii. The circles visualize the predicted radii for all computed Dipid designs. Experimentally tested designs (XS, S, M, L, XL and XXL) are shown as thick, coloured lines. **d**, Mean Dipid structures simulated with oxDNA, alongside representative negative-stain TEM images of Dipid monomers and a planar assembly.

In the present work, we sought to combine the nanoscale precision enabled by sequence-programmable building blocks with the benefits of membrane-like architectures (Fig. 1a). Thus, we developed an approach based on DNA origami subunits that we termed Dipids, whose architecture and interactions are inspired by the structure of lipids. Dipids are designed as isotropic sticky discs^26^ that bind to their neighbours through a network of flexible DNA strands with weak hybridization interactions. We demonstrate that Dipids enable the assembly of diverse membranous structures, like planar membranes, containers and tubes, which range in size from the nanometre to the micrometre scale. Notably, each structure self-assembles from a single, structure-specific Dipid monomer type that is rationally designed from the same fundamental base unit. We expand the Dipid concept by demonstrating that heterogeneous mixtures of Dipids with distinct characteristics function as versatile membrane patchy particle systems that reproduce key lipid membrane features, such as phase separation and differential curvature. By developing a diverse set of structural and functional Dipids, we finally create a modular toolbox for the realization of functional Dipid-based containers.

## Design principles for lipid-inspired DNA origami

We hypothesized that a minimal set of properties is necessary for a non-amphiphilic-membrane-forming monomer and implemented these using DNA origami^27,28^ (Fig. 1b). Among other DNA-based approaches^29,30^, DNA origami enables the programmable assembly of custom-shaped structures by folding a single-stranded DNA (ssDNA) scaffold with rationally designed staple strands, and it is, thus, ideally suited for realizing such a monomer.

To achieve lipid-like isotropic interactions within a membrane, we selected as our starting point a rotationally symmetric, well-characterized DNA barrel structure with a diameter of 30 nm that was developed by Wickham et al.^31^. The homomeric self-assembly of this monomer into higher-order structures offers several kinetic advantages. Its circular geometry promotes hexagonal packing, which is predicted to assemble more rapidly than alternative lattice configurations^32^. The rotational symmetry of the monomers enhances the effective binding, as successful intermonomer bonds can form irrespective of their rotational orientation. Moreover, compared with previously reported DNA origami structures used for large-scale assemblies^14^, our barrel design features thin walls that provide structural compliance and accommodate local geometric strain.

**Extended Data Fig. 1:**
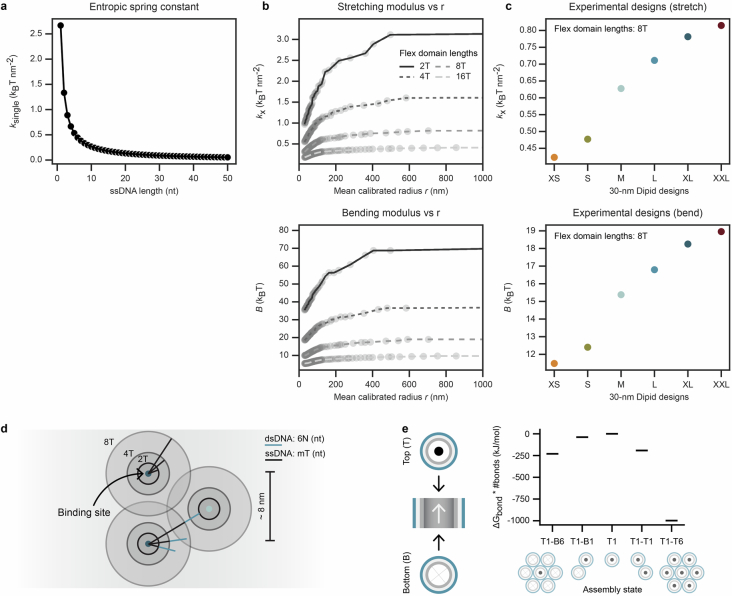
Additional Dipid design properties and considerations. **a**, Entropic spring constant *k*_single_ of a single ssDNA overhang as a function of its length, computed with the freely-jointed-chain model assuming a Kuhn length *l*_*k*_ = 1.5 nm. **b**, Predicted stretching modulus *k*_*x*_ and bending modulus *B* of the Dipid dimer interface as a function of calibrated container radius *r*. Grey tones and dash patterns encode ssDNA flex domain lengths (2T, 4T, 8T, 16T). Moduli were computed with the framework of Videbaek et al.^72^ adapted to Dipid dimensions. The original study found good agreement for *B* but a systematic overestimate of *k*_*x*_, expected here as well. **c**, Estimated stretching and bending moduli for the six experimentally tested container designs (XS-XXL). Source code for all calculations is available at our GitHub repository. **d**, Interaction cross-section for binding strands on the origami surface. Binding sites (origins of binding strands) are shown as filled dark or light blue dots for the two binding strand groups. Shaded areas denote the maximal delocalization for flex domains with 2T, 4T, or 8T. Teal linker extensions indicate the contour length of minimal binding strands with 6-nt sticky sequences, using inter-base distances of 0.63 nm for ssDNA and 0.34 nm for dsDNA. **e**, The preference for 2D growth can be rationalized via Dipid-Dipid binding energies. For each state, we estimated *ΔG* by multiplying the average linker hybridization energy (38.38 kJ/mol) by the number of sterically feasible bonds between a central upward monomer and its nearest neighbours. Monomers were assumed to bind mainly through the binding strand groups containing three binding strands. In the T1-T6 configuration, where 3 × 2 compatible strands are shared between three connected monomers, only two of each type can hybridize simultaneously. We therefore counted these as $\frac{2}{3}$ of a full bond.

**Extended Data Fig. 2:**
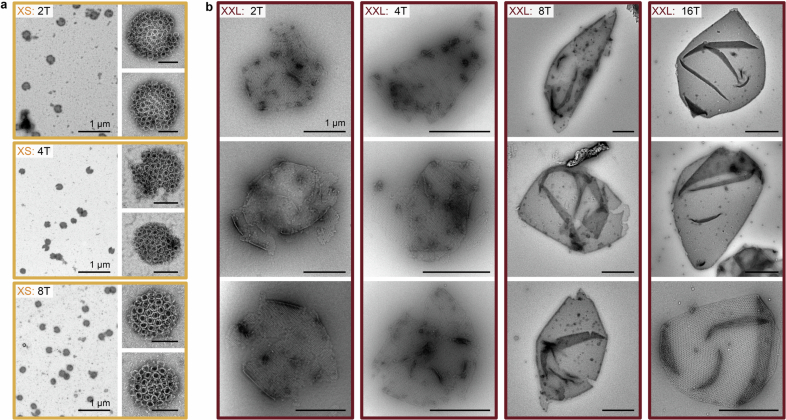
Longer flex domains lead to structurally more intact containers under the TEM. **a**, Unpurified XS containers formed by Dipid designs with constant flex domain lengths of 2T, 4T, or 8T. Scale bars: 100 nm unless specified otherwise. **b**, Purified and annealed XXL containers formed by Dipid designs with constant flex domain lengths of 2T, 4T, 8T, or 16T. With increasing flex domain length, the containers appeared more intact under the TEM. We attribute this to improved resistance to deformation upon collapse on the grid, resulting from longer flex domains and thus higher flexibility and stretchability. Scale bars: 1 µm.

**Extended Data Fig. 3:**
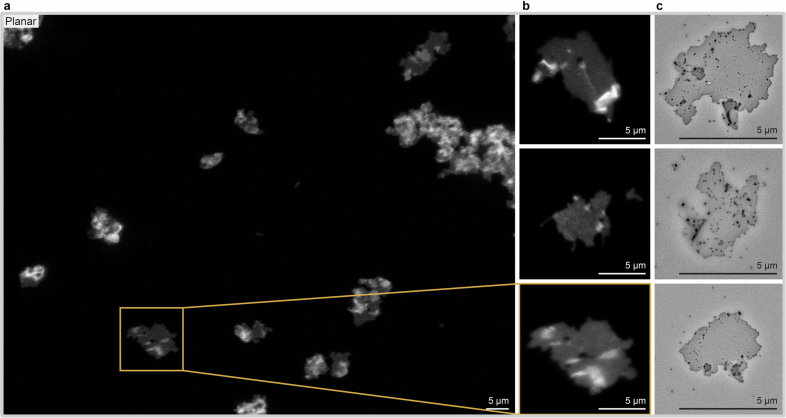
Structures formed by Dipids without designed curvature and with an 8T flex domain. **a,b**, Confocal microscopy overview and details of mostly planar assemblies. Dipids were labelled with an Atto488 dye, conjugated to an internal staple. **c**, TEM micrographs of planar assemblies.

We implemented these design criteria by incorporating functional domains into ssDNA strands protruding from the surface of the barrel (Fig. 1b). Each monomer carries 30 binding strands distributed in alternating groups of two and three, which form two overlaid sixfold-symmetric interaction patterns. We introduced an oligo-dT ‘flex’ domain to encode intermonomer flexibility (Extended Data Figs. 1 and 2) and prevent the accumulation of strain. Combined with the dense binding-strand coverage, strand flexibility enables a nearly continuous intermonomer interaction profile (Extended Data Fig. 1d). Next, we introduced ‘sticky’ domains, each composed of a weak palindromic binding sequence to ensure reliable in-plane assembly. The rotationally symmetric binding profile and the associated random relative rotations within higher-order assemblies are expected to average out minor asymmetries in the core monomer design. We expect the weak binding affinities and staggered binding-strand positions of the sticky domain will open a temperature regime for self-assembly in which undesired three-dimensional (3D) aggregates are unstable, whereas correct two-dimensional (2D) membranes can form stably (Extended Data Fig. 1e). Based on these design considerations, Dipids can be readily programmed to generate extended 2D membranes (Extended Data Fig. 3).

Finally, we introduced an optional ‘curvature’ domain by strategically extending some of the flex domains with variable-length segments, resulting in cone-shaped Dipids that promote global membrane curvature (Fig. 1b). One of our central hypotheses is that the self-assembly of closed membranes can be induced by introducing local curvature to subunits, thus bypassing the binding rules and symmetry-governed Caspar–Klug principles of capsid construction. Notably, curvature and binding domains can be modified independently to allow customizable interactions and non-uniform curvature profiles.

To enable rapid prototyping, we established an automated design pipeline for generating Dipid variants. Using a simple geometric model and a small set of DNA-sequence boundary conditions, combined with a worm-like chain polymer approximation, we systematically produced 74 unique monomer designs characterized by their predicted radius of curvature *r* (Fig. 1c). Finally, we verified that our design modifications correctly preserve the DNA origami folding and enable 2D assembly (Fig. 1d).

## Containers self-assemble from a single Dipid type

**Fig. 2:**
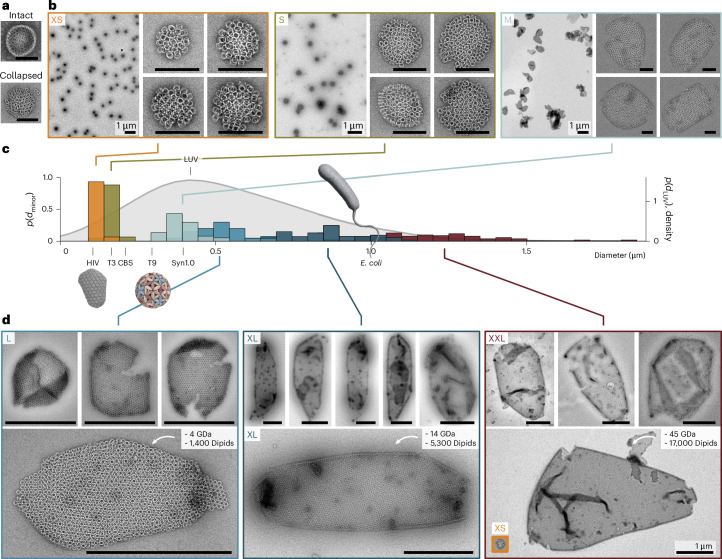
Dipid variants assembling into small, medium and cell-sized containers. **a**, Example TEM images of an intact spherical container supported by excess EM stain, as well as a collapsed and ruptured container flattened on the TEM grid. **b**, Representative TEM overviews and detailed examples of containers formed from the XS, S and M Dipid designs. **c**, Probability distributions of the minor 3D diameters (*d*_minor_) of the containers. For comparison, the dimensions of selected biological and state-of-the-art synthetic compartments are also listed. The dimensions were extracted from refs. ^14,33–36,49,50^. The HIV capsid is a render of Protein Data Bank entry 3J3Q (ref. ^34^). The T9 model was kindly provided by the Dietz group. Distributions of large unilamellar vesicles are shown as a kernel density estimate. Sample sizes: *n*_XS_ = 59, *n*_S_ = 119, *n*_M_ = 37, *n*_L_ = 20, *n*_XL_ = 53 and *n*_XXL_ = 100. The data represent independent container measurements from one assembly reaction per Dipid variant. **d**, TEM images of selected containers formed from the L, XL and XXL Dipid designs. The XS container shown as an inset in the large XXL example is displayed at scale. The container mass and the number of assembled Dipids were estimated from the container surface areas. Scale bars: 200 nm (**a**), 200 nm (**b**, unless specified otherwise), 1 μm (**d**). The T9 capsid model in panel **c** was adapted with permission from ref. ^14^, Nature. LUV, large unilamellar vesicle.

We first sought to validate that an isotropic cone angle alone is sufficient to drive the self-organized formation of the topological defects required for closed container assembly while preserving diameter control. Transmission electron microscopy (TEM) images of six experimentally realized Dipid variants (XS, S, M, L, XL and XXL) demonstrate robust self-assembly into increasingly larger closed structures (Fig. 2a,b,d) and reveal a narrow size distribution compared with the large unilamellar vesicles commonly used in synthetic biology^33^ (Fig. 2c).

**Extended Data Fig. 4:**
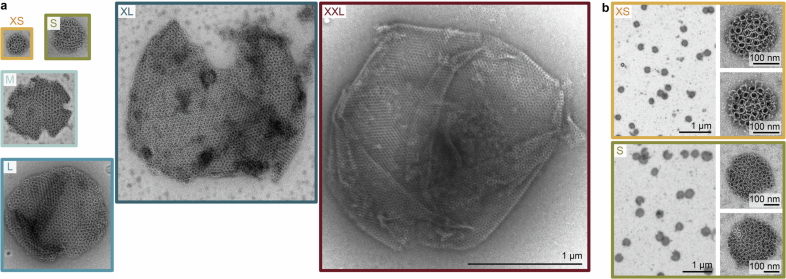
Containers already forme during the DNA origami folding process. **a,b**, TEM images of collapsed containers formed before purification or re-annealing. **a**, All images are displayed at the same pixel size. **b**, TEM overview showing the container yield and a zoom-in of unpurified, collapsed XS and S containers.

The XS and S Dipid variants formed the targeted spherical containers with a high yield, as shown in the representative TEM overview images in Fig. 2b. Container assembly occurred already during the initial DNA origami folding. Subsequent purification and reannealing did not notably improve the yield of the smaller XS and S containers (Extended Data Fig. 4). With mean diameters of $${\overline{{{d}}}}_{{\rm{XS}}}=119\pm 14\,{\rm{nm}}$$ and $${\overline{{{d}}}}_{{\rm{S}}}=166\pm 16\,{\rm{nm}}$$, the XS and S container dimensions are like those of large protein compartments, such as HIV capsids and *β*-carboxysomes^34^^,35^. For larger container designs, we observed an increasing fraction of non-spherical morphologies. M and L containers (minor container axes $${\overline{{{d}}}}_{{\rm{M}}}=396\pm 51\,{\rm{nm}}$$ and $${\overline{{{d}}}}_{{\rm{L}}}=513\pm 69\,{\rm{nm}}$$) considerably exceed the size of the largest DNA origami container reported so far (*d* ≈ 300 nm), which was constructed following the design principles for icosahedral capsids^14^. An average L container is already large enough to engulf a Syn1 cell, a minimal synthetic bacterium with a lipid-bilayer-based cell membrane^36^. Finally, our XL and XXL containers ($${\overline{{{d}}}}_{{\rm{XL}}}=862\pm 119\,{\rm{nm}}$$ and $${\overline{{{d}}}}_{{\rm{XXL}}}=1.2\pm 0.2\,\upmu {\rm{m}}$$) reach and even exceed the dimensions of *Escherichia* *coli* cells. Notably, transforming an S Dipid into an XXL Dipid requires no further design effort due to our automated pipeline but just the exchange of 24 binding strands with a cost of approximately $160.

**Extended Data Fig. 5:**
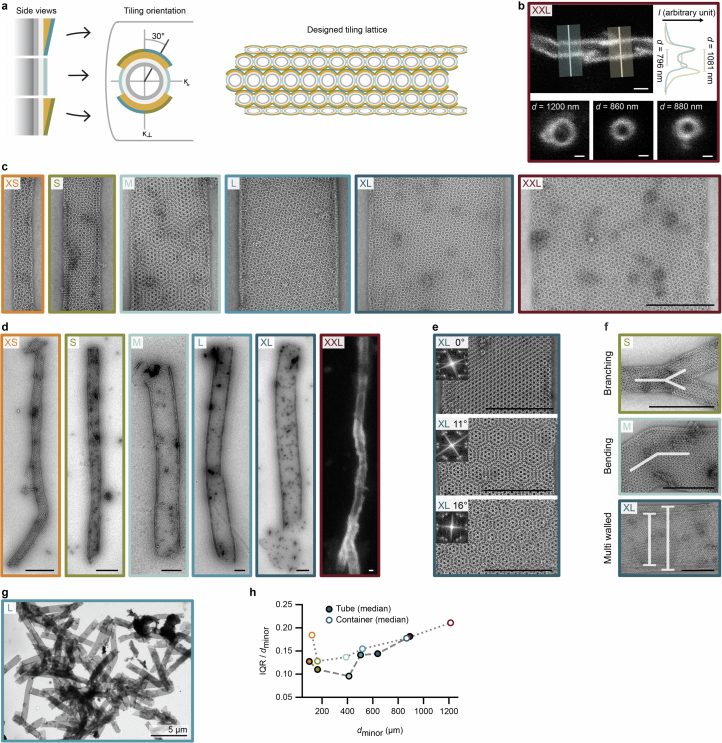
Formation of open tubes with programmable diameters. **a**, Monomer design for the formation of tubes with programmable diameters. Differently coloured sticky layers (blue, light blue, and green) indicate binding strand sets with orthogonal sticky domains. **b**, Close-up confocal microscopy *z*-stack projections. In solution, tubes may be oriented parallel to the plane, which visualizes the variation of the cross-section along a tube’s length (top row), or orthogonal to the imaging plane, displaying circular cross-sections (bottom row). The inset shows intensity plots along the indicated lines. **c**, TEM micrographs of tubes with increasing diameters from left to right, shown at the same magnification. **d**, Exemplary overview images showing complete tube morphologies. The XXL-tube image is a confocal microscopy z-stack maximum projection. **e**, Gallery of Moiré patterns acquired from a single sample of XL tubes imaged on the same TEM grid. Insets show fast Fourier transforms (FFT) and the corresponding relative orientation angles between the upper and lower tube walls. **f**, Selected TEM micrographs highlighting several of the observed growth defects such as branching, bending coupled with diameter changes, and growth of multi-layered tubes. Scale bars: 500 nm. **g**, Example TEM micrograph of clustered L tubes in a re-annealed sample. Tubes, or other extended structures, might cluster due to electrostatic attraction, naturally occurring Dipid dimers, misfolded Dipids, or tube-growth defects. **h**, Diameter distribution widths (interquartile range, IQR), normalised by the median Dipid design diameters, for tube and container data as shown in Fig. 3g.

**Extended Data Fig. 6:**
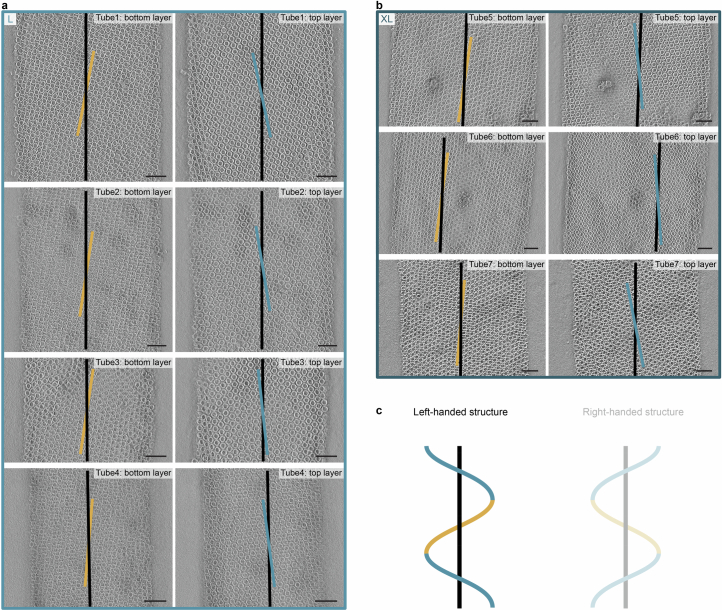
Tomograms of tubes reveal a preferred left-handed helicity. **a,b**, Tomogram slices showing the bottom and top of L (a) and XL (b) tubes. In achiral tubes, the Dipid tiling forms ‘lines of Dipids’ parallel to the tube axis (black line). In chiral tubes, these lines are tilted (orange and blue lines), indicating helicity. **c**, Schematics illustrating left- and right-handed helicity, and the corresponding tilt of the central line relative to the tube axis. Scale bars: 100 nm.

To demonstrate the extensibility of the Dipid system to non-uniform curvatures and programmable non-isotropic interactions, we reprogrammed the Dipid interaction patterns to form open tubes (Extended Data Fig. 5 and Supplementary Note 1). Such designs reliably form tubular structures, with a diameter control comparable to that of our containers. We note that all tubes reconstructed from TEM tilt series exhibited left-handed helicity (*n* = 7), possibly resulting from subtle asymmetries in the binding affinities of the non-isotropic interaction patterns in the Dipid tube designs (Extended Data Fig. 6).

**Extended Data Fig. 7:**
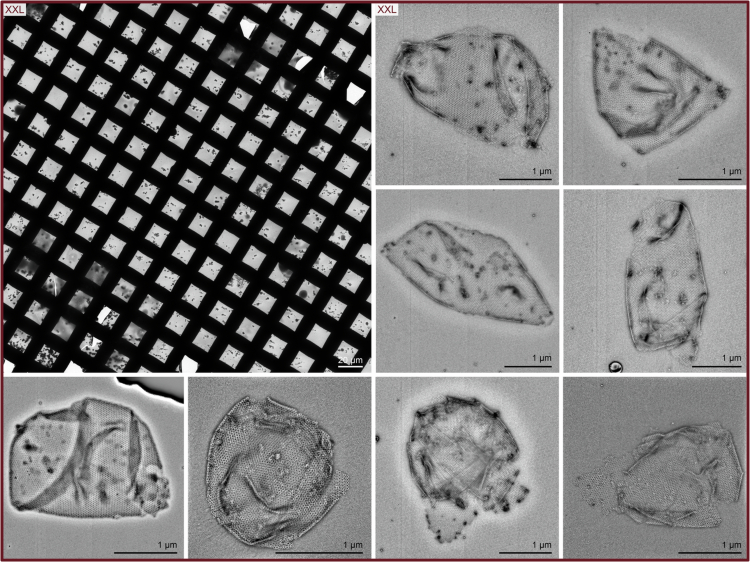
Production of XXL containers within 28 h. Representative TEM grid overview, next to collapsed XXL containers produced with a 4 h folding protocol, followed by filter purification and a 24 h annealing protocol.

In the initial experiments, we annealed tubes and containers using a multi-day annealing protocol, which is typical for large DNA origami assemblies^14,15^. However, we later found that a much shorter protocol (4 h folding and 24 h annealing) is sufficient to yield assemblies of comparable quality for the XXL container design (Extended Data Fig. 7).

**Extended Data Fig. 8:**
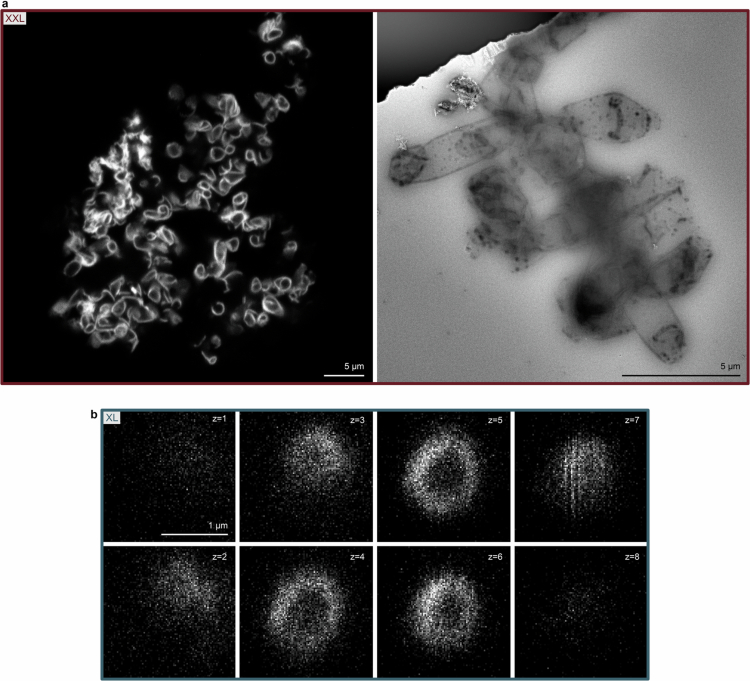
Confocal and TEM images of an extended membrane cluster and an isolated container. **a**, The Dipid membrane clusters were formed by XXL design Dipids and show non-uniformly curved structures, including partially open ones. **b**, z-slices of an XL container (*Δz* = 0.3 µm), highlighting its hollow structure. Dipids were labelled with an Atto488 dye, conjugated to an internal staple.

Although we rarely observed unspecific 3D aggregation, we noted an increased tendency to form extended, non-uniformly curved 2D membranes with decreasing membrane curvatures (Extended Data Fig. 8). Such behaviour is consistent with our intended emulation of lipid assembly, which is inherently associated with structural polydispersity.

## Structural analysis of the containers

**Fig. 3:**
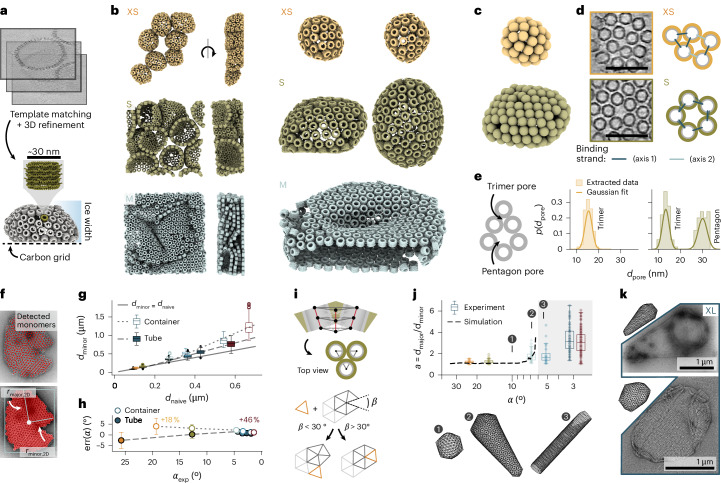
Structure analysis and simulations. **a**, Reconstruction pipeline for Dipid membranes from cryo-ET data and expected deformations due to grid interactions and limited ice width. **b**, Left: Reconstructions of container and membrane aggregates (XS, S and M). Right: Examples of reconstructed containers (XS and S) and container fragments (M). **c**, Containers with spheres representing each Dipid, highlighting local packing geometries. **d**, Right: 15-nm-thick tomographic slices of two pentagonal disclination types. Left: Possible underlying binding geometries. Semitransparent bonds indicate the preferred binding angles. **e**, Pore-size distributions for trimeric pores in a hexagonal lattice and pentagonal disclinations from XS and S containers, measured as the largest inscribed circle. **f**, Pipeline for detecting containers and fitting ellipses to the container areas to extract their principal radii *r*_minor,2D_ and *r*_major,2D_. **g**, Distribution of 3D container and tube diameters from collapsed 2D assemblies, using *d*_minor_ = (4*r*_minor,2D_)/π. Diameters predicted by the naive model *d*_naive_ are displayed on the *x* axis. Dotted lines show heuristic calibration fits to container and tube data in the two linear regimes. The different error scaling for tubes may reflect their non-isotropic curvature and binding affinity or the reduced number of binding strands (Extended Data Fig. 5). Boxes show medians and interquartile ranges (IQRs). Whiskers span 1.5 × IQR, and outliers are shown as points. Sample sizes for container and tube data: *n*_XS_ = 59/83, *n*_S_ = 119/216, *n*_M_ = 37/71, *n*_L_ = 20/83, *n*_XL_ = 53/112 and *n*_XXL_ = 100/74. The data represent independent container measurements from one assembly reaction per Dipid variant. **h**, Angle error between design (*α*_naive_) and experiment (${\alpha }_{\exp }$) for *d*_minor_ data in **g** (median ± s.d.). **i**, Assembly rules of an HIV-inspired assembly simulation (compare ref. ^40^), adapted to represent Dipid dimensions. The opening angle between two edges is defined as *β*. **j**, Experimental aspect ratio distribution *a* = *d*_major_/*d*_minor_ from data in **g**, overlaid with mean aspect ratios from simulations using *α*_naive_ and ${\alpha }_{{\rm{sim}}}$. Simulations with ${\alpha }_{{\rm{sim}}} < {6.3}^{\circ }$ produced only tubes (grey). See Supplementary Table 1 for simulation counts per *α*_sim_. Boxes defined as in **g**. **k**, TEM images of XL containers showing two exemplary morphology variants, comparable to simulations with ${\alpha }_{{\rm{sim}}}={7.2}^{\circ }$ and 5% random monomer placement. Scale bars: 100 nm (**d**).

We used cryo-electron tomography (cryo-ET) to reconstruct membrane structures from tomograms by template-matching^37,38^, followed by manual selection of containers and 3D refinement (Fig. 3a). XS, S and M Dipid variants self-assembled into closed, hollow containers but also into intricately folded membrane aggregates (Fig. 3b). A local analysis of monomer packing revealed a surprisingly wide range of structural arrangements accessible through Dipid assembly. In addition to symmetric and asymmetric pentagonal defects, XS containers also exhibit amorphous monomer organization (Fig. 3c,d). Together, these findings show that the Dipid design principles enable the autonomous formation of disclinations, which effectively resolves the curvature-driven packing constraints.

**Extended Data Fig. 9:**
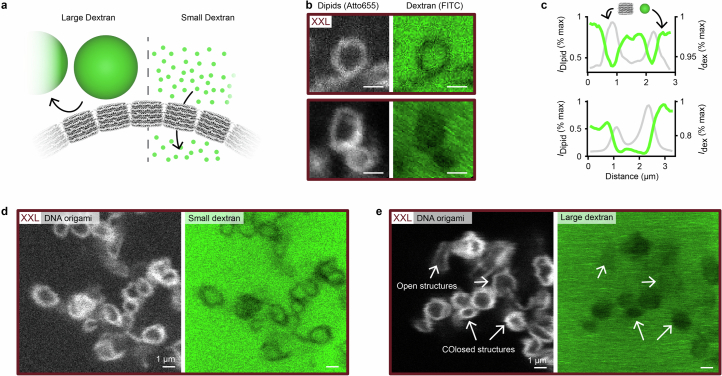
Closed Dipid structures show size-selective permeability. **a**, Scheme of a Dipid membrane permeation experiment using fluorescently labelled dextrans of different sizes (*M*_*w*_(small dextran) ≈ 4 × 10^3^ g mol^−1^, *d*_hydro_ ≈ 4 nm; *M*_*w*_(large dextran) ≈ 2 × 10^6^ g mol^−1^, *d*_hydro_ ≈ 54 nm^73^). **b**, Confocal microscopy images of Dipid container clusters after the addition of small or large dextran solutions as in the scheme in panel **a**. The Dipid channel (labelled with Atto655) is shown in gray, and the FITC-labelled dextran channel is shown in green. Scale bars: 1 µm. **c**, Normalised and smoothed fluorescence intensity profiles extracted from the images in panel b. Gray and green lines show Dipid membrane and dextran intensities, respectively. **d,e**, Representative confocal images of XXL container and membrane clusters in solutions of differently sized dextrans. Notably, structures appearing closed in one plane may be open in others. In panel **d**, the small dextran signal inside closed structures is comparable to the ambient dextran signal. In panel **e**, some containers in the container and membrane clusters show a lower dextran signal in their lumen, while other, presumably open structures show ambient level dextran signals, comparable to containers in the small dextran solution.

Based on the container reconstructions, we next determined the approximate pore sizes between monomers in their close-packed state (*d*_trimer_(XS) = 15 ± 2 nm and *d*_trimer_(S) = 13 ± 2 nm) and within pentagonal disclinations (*d*_pentagon_(S) = 30 ± 2 nm), assuming high salt concentrations and correspondingly small electrostatic screening lengths (Fig. 3e). With the designed internal Dipid pore size *d*_internal_ ≈ 19 nm, pentagonal pores should represent the limiting pore size in the absence of occasionally missing monomers (*d*_defect_ > 30 nm). Indeed, permeability experiments with dextran showed size-selective permeability for closed structures within XXL container and membrane clusters (Extended Data Fig. 9).

We next extended our analysis to larger containers using TEM imaging. Container dimensions were extracted by automated ellipse fitting to collapsed containers, yielding the corresponding 3D container diameters *d*_major_ and *d*_minor_ (Fig. 3f). We first compared the experimentally determined *d*_minor_ to the naive Dipid model predictions. Although the two smallest container variants closely matched their designed diameters, larger containers systematically exceeded their predicted sizes, displaying a nearly linear deviation from the naive model. A similar deviation was observed for tubes but with a different scaling behaviour, which required separate heuristic calibration curves to refine the Dipid design predictions (Fig. 3g). The diameter variability generally increases with increasing target diameter, with exceptions for small-diameter structures (Extended Data Fig. 5h). The increasing deviation between designed and experimental diameters for both geometries probably reflects the limited applicability of polymer-based approximations for short ssDNA binding strands. Additionally, design-related cone angle inaccuracies become disproportionately impactful at larger radii, amplifying minor systematic errors in our monomer model (Fig. 3h).

## Simulation of container growth

The progression of shapes formed by Dipid assemblies—from spherical (XS and S) and fullerene-like (M and L) to closed tubular structures (XL and XXL) (Fig. 2)—closely mirrors the structural transitions observed in simulations originally developed to explain the polymorphism of HIV capsids. In such simulations, decreasing the monomer cone angle *α* drives a shift in morphology from spherical to open tubular structures^26,39,40^. To allow a better comparison, we adapted one such simulation^40^ to match the dimensions of our Dipid structures while preserving its original assembly rules (Fig. 3i). We found that above a certain cone angle (*α* ⪆ 6.3°), the simulated morphologies closely resemble their experimental counterparts in terms of the container aspect ratio (*a* = *d*_major_/*d*_minor_; Fig. 3j). With a small percentage of monomers placed randomly during assembly (5%), the simulation also reproduces the experimentally observed polymorphism at constant *α* (Fig. 3k). However, by design, the simulation cannot predict the amorphous membrane architecture of XS containers. Also, although the simulation predicts a transition to open tubes for small *α*, we experimentally found the continued formation of closed structures.

## Dipids as membrane-forming patchy particles

**Fig. 4:**
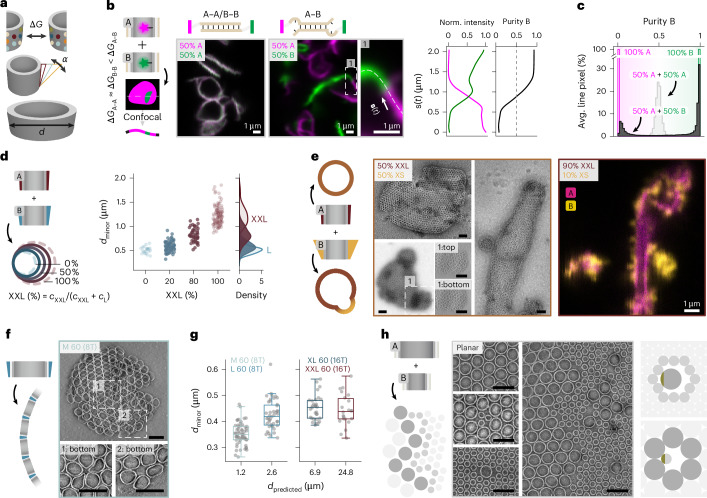
Dipids as membrane-forming patchy particles for modelling complex self-assembly phenomena. **a**, Schematic of tunable Dipid properties: binding affinity (Δ*G*), cone angle (*α*), connection flexibility and diameter (*d*). Each property is independently adjustable to modulate membrane behaviours. **b**, Domain formation in Dipid membranes assembled from planar Dipids with identical (A–A) versus partially complementary (A–B) binding strands. Domain purity was computed along the membrane coordinates of confocal images ($\mathbf{s}(t)$). **c**, Distribution of membrane skeleton pixel purities, obtained from skeletonized averaged membrane pixels (*n* = 100 images) $\mathbf{s}(t)$. **d**, Tunable control of container size through co-assembly of Dipids with different preferred curvatures (L and XXL variants). Diameters were extracted from TEM images. Distributions are shown as kernel density estimates. **e**, Curvature-driven domain formation in co-annealed XS and XXL Dipid samples leading to intermediate diameter containers and complex, non-homogeneous curved structures. Confocal imaging of fluorescently labelled Dipids (XS-Atto488 and XXL-Atto655) highlights domain formation. **f**, Left: Schematic of self-assembled containers from larger (60-nm) Dipids. Right: TEM image of a 60-nm Dipid container and tomogram slices showing local monomer deformation in pentagonal defects, enabled by increased monomer flexibility. **g**, Diameter distributions of containers formed from four 60-nm Dipid variants with increasing predicted diameters and differing flex domains (8T and 16T). Boxes show medians and IQR. Whiskers extend to 1.5 × IQR. Data represent independent container measurements from one assembly reaction per Dipid variant (*n*_M60_ = 57, *n*_L60_ = 43, *n*_XL60_ = 32 and *n*_XXL60_ = 22). **h**, TEM micrographs of defects and domain boundaries found in membranes of co-annealed 60-nm and 30-nm planar Dipids, next to a schematic depicting the possible binding sites (green) of undeformed Dipids in the lattice of the other Dipid. Scale bars: 100 nm unless specified otherwise. Avg., averaged; Norm., normalized.

To enable the systematic exploration of more complex membrane self-assembly mechanisms, we expanded the Dipid framework into a versatile membrane-forming patchy particle system (Fig. 4a). Many sophisticated functions of biological membranes arise from spatial organization governed by local variations in molecular affinity, curvature and composition^41^. In binary Dipid mixtures, we can independently tune these parameters, thus leveraging the inherent programmability of the Dipid monomers. To model affinity-driven domain formation reminiscent of lipid rafts^41^, we introduced specific intermonomer affinity differences using partially complementary interaction domains between two planar Dipid variants A and B (Fig. 4b). Confocal microscopy revealed clear domain formation in the A–B system, characterized by the nearly complete demixing of fluorescently labelled Dipids into distinct phases, including A-rich to B-rich transitions at domain boundaries (Fig. 4b). A membrane profile analysis confirmed the high domain purity, with clear segregation of monomer types (Fig. 4c).

To investigate curvature-driven membrane organization reminiscent of biologically important processes such as clathrin-mediated budding^41^, we co-assembled Dipid variants with distinct cone angles, representing different intrinsic curvature preferences (Fig. 4d). Binary mixtures with moderate curvature differences (for example, L and XXL) reliably produced containers of intermediate sizes, with diameters tunable through the mixing ratio. By contrast, the co-assembly of Dipids with larger curvature mismatches (for example, XS and XXL) yielded complex morphologies, including containers with locally distinct curvature domains and capped tubular structures. Confocal microscopy confirmed the partial separation into XS-rich and XXL-rich domains (Fig. 4e) caused by curvature-driven demixing.

To investigate how membrane-embedded structures influence membrane organization—analogous to the mutual ordering effects between membrane proteins and surrounding lipids^42^—we extended the Dipid design to a larger, 60-nm barrel architecture^31^. This variant retained the core Dipid assembly logic while incorporating enhanced isotropic interactions and increased structural flexibility. We first confirmed that the larger Dipids could still form closed containers (Fig. 4f), although they exhibited pronounced deformability and a growing mismatch between predicted and experimental diameters with increasing flex-domain length (8T to 16T) (Fig. 4g). To probe membrane behaviour in mixed systems, we co-assembled 30-nm and 60-nm Dipids carrying identical binding domains. These binary membranes displayed clear size-dependent partial demixing, characterized by isolated lattice defects, enriched domains of uniform monomer size and sharply defined interface boundaries (Fig. 4h).

## Plug and play: a Dipid-based design framework

**Fig. 5:**
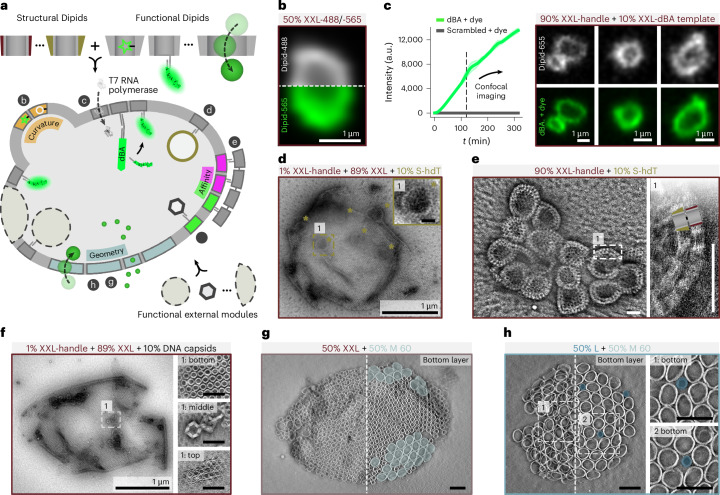
Establishing a Dipid-based design framework by integrating structural and functional modules. **a**, Schematic illustrating the integration of structurally distinct and functional Dipids, as well as externally developed modules, within a single multifunctional Dipid container. Letters b–h next to specific modules indicate the corresponding figure panels that demonstrate these implementations. **b**, Confocal image of a homogeneously mixed membrane container formed by two Dipid modules carrying distinct small molecules, demonstrating the unperturbed assembly of internally modified Dipids. **c**, Implementation of an IVT module using the fluorescent dBA. Bulk measurements and confocal measurements after 2 h show sustained dBroccoli transcription and membrane localization, with a scrambled template showing no signal increase (bulk data shown as mean ± s.d. for *n* = 3 technical replicates). **d**, Encapsulation of preassembled, high-disassembly-temperature S (S-hdT) Dipid containers within larger XXL Dipid containers. Encapsulated containers are indicated by green stars. The inset shows a zoom-in of the marked position **e**, Formation of Dipid bilayers from S-hdT-binding XXL monomers around clusters of S-hdT containers. **f**, Left: Encapsulation of externally developed DNA origami capsids inside Dipid compartments through membrane-localization modules. Right: Tomogram slices confirm successful internal localization. **g,h**, Co-assembly of 30-nm Dipids with 60-nm Dipid pores into containers, with partial demixing leading to 60-nm pore rafts marked in light blue (**g**) and preferred 30-nm Dipid localization in 60-nm pentagonal disclinations of 60-nm Dipid pore-rich containers marked in blue (**h**). Scale bars: 100 nm unless specified otherwise.

We next expanded the structural Dipid framework with programmable functional modules, paving the way for applications in synthetic biology and soft cell-scale robotics (Fig. 5a). The simplest route to creating functional Dipids is to decorate the interior of the monomer barrels with functional molecules. Given the monomer’s inner volume of approximately 5,000 nm^3^, Dipids may be modified with various functionalities—nanoparticles, proteins, nucleic acids or small molecules—as ‘plug-in’ functions without compromising membrane assembly (Fig. 5b).

Alternatively, functional molecules can be conjugated to the lower or upper side of a Dipid, pointing inwards or outwards. As a proof of principle, we developed an in vitro transcription (IVT) module using the fluorogenic dBroccoli aptamer (dBA), along with a dBA capture and membrane localization module (Fig. 5c). To this end, on average every tenth Dipid was engineered to present a transcription template for dBA, which was extended with a 20-nt-long capture sequence. The remaining Dipids were modified to display inward-facing capture strands to which the capture sequence of the aptamer could hybridize. A key enabler of this system is the semipermeability of closed Dipid membranes, which allows postassembly delivery and a constant supply of small molecules and active components. This feature offers a distinct advantage over lipid-vesicle-based systems, where the sustained delivery of components to the lumen can be limited. For our IVT module, externally added T7 RNA polymerase can enter the compartments through the pores and transcribe the membrane-immobilized templates, which is followed by membrane localization of the RNA products (Fig. 5c).

A third strategy for functionalizing Dipid containers is to embed preassembled structures as modular subcompartments, mimicking biological organelles. We demonstrate this by encapsulating preformed S containers (S-hdT)—adapted to have a high disassembly temperature and three XXL binding sites—into XXL containers containing 1% S-hdT-binding XXL Dipid modules (Fig. 5d). Notably, in experiments where we equipped 100% of the XXL Dipids with an S-hdT binding module, we observed XXL Dipids forming bilayers around S-hdT container clusters (Fig. 5e). Some applications may require specialized compartments beyond what the Dipid architecture alone can provide. As an example, we used Dipid membrane-localization modules to encapsulate preformed (non-Dipid) DNA origami capsids^14^ as subcompartments (Fig. 5f).

**Extended Data Fig. 10:**
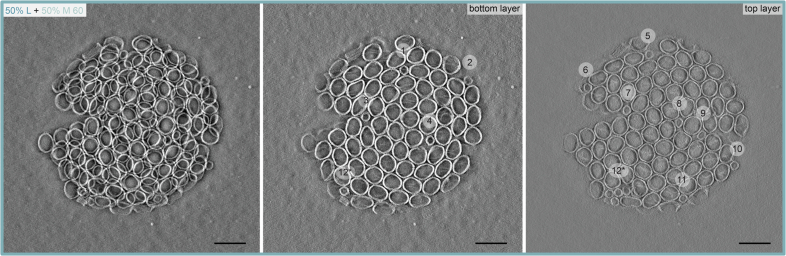
30-nm Dipids localize in pentagonal disclinations of 60-nm Dipid containers. TEM micrograph and tomogram reconstructions of a container formed by co-annealing 30-nm Dipids (L) with 60-nm Dipids (M). The container contains 17 30-nm Dipids, 11 of which occupy pentagonal disclinations prior to container collapse. A cluster of six 30-nm Dipids, marked as ‘12*’, forms a more complex disclination. Scale bars: 100 nm.

A potentially even more powerful strategy is the direct integration of functional modules with diverse shapes and capabilities into the membrane. For instance, by repurposing the 60-nm Dipid variant as a ‘large-pore’ module and co-annealing it with container-forming 30-nm Dipids, we demonstrate that size-mismatched modules can be stably integrated into Dipid membrane containers, provided their interaction domains are compatible (Fig. 5g,h). As anticipated, the geometrically mismatched Dipid modules partially demixed into containers enriched in either 30-nm or 60-nm Dipids. Although 60-nm Dipids formed rafts within 30-nm Dipid-rich containers, we observed that pentagonal disclinations in predominantly 60-nm containers were frequently occupied by individual 30-nm Dipids, indicating a strategy for the deliberate placement of functionalities at lattice defects (Extended Data Fig. 10).

## Conclusion

With the Dipid framework, we introduce a lipid-inspired approach for generating large super-assemblies from DNA origami subunits that are capable of forming extended 2D membranes, tubes and closed containers with controllable sizes ranging from 100 nm to several micrometres. With a mass of over 40 GDa, our XXL Dipid containers are among the largest demonstrated with the tools of DNA nanotechnology to date.

Our framework represents a departure from traditional approaches in the field, which have focused either on the realization of super-assemblies from precisely defined, structurally rigid monomeric subunits or on the formation of microgels, lipid vesicles or liquid droplets through phase separation. Using only a single, highly symmetric DNA origami monomer unit with flexible and redundant binding interactions, our method enables the simple and cost-effective generation of DNA origami superstructures with adaptable properties. As demonstrated in this work, our larger super-assemblies easily reach the size of small unicellular organisms or those of large unilamellar vesicles made from phospholipids.

Dipids are a class of membrane-forming patchy particles that enable the exploration of self-assembly and self-organization strategies in an unprecedented manner. They are ideally suited as an experimental system that is programmable and well-controlled enough to allow the direct implementation of theoretical models in real-world experiments. In this work, we have demonstrated that concepts from soft matter biophysics—such as demixing, raft and domain formation, and curvature-induced membrane organization—can be directly implemented in experiments through the rational design of the building blocks. Notably, using the Dipid patchy particle system, it is possible to realize new constructs, such as frustrated assembly through topological defect engineering and the formation of finite-sized superstructures^43^.

Our Dipid framework opens up applications based on an alternative approach to the compartmentalization of synthetic biological systems in bottom-up biology. Compared with vesicles, Dipid-based compartments offer several potential advantages, including customizable size and curvature, structural stability, and tunable porosity. We have, thus, established a versatile toolbox for the rational and hierarchical design of functional compartments that allow for the spatial organization of cell-free biochemical systems. These compartments can be assembled from monomers carrying different chemical or mechanical functionalities, such as enzymes, gene templates, molecular motors or inorganic components. Further, the integration of modules for sensing and signalling^44^, computation^45^, bioproduction^46^ or locomotion is expected to enable the programming of these compartments as soft, cell-scale robotic systems.

## Methods

### Design of sticky domains using NUPACK

The orthogonality of sets of sticky domains was evaluated using the NUPACK web service (model = dna04, temperature = 25 °C, maximum complex size = 2 and strand concentration = 1 M). The hybridization free energies were computed with the NUPACK Python package^51^ (material = ‘dna’, celsius = 20 °C, sodium = 0.05 M and magnesium = 0.018 M).

### Length calculations of binding strands for specific curvatures

We calculated the physical length of binding strands *l*_strand_ using constant flex-domain lengths for all strands in a given design (mT = 2T, 4T, 8T or 16T), while varying the sticky-domain lengths (xN = 4, 6 or 8N) and curvature-domain lengths (nT = 0–52T). We used base-to-base distances of 0.63 nm per base for ssDNA domains^52^ and 0.34 nm per base for double-stranded DNA domains to calculate the respective contour lengths, *L*_c_. The persistence length of ssDNA *L*_p_ was set to 1.5 nm (ref. ^52^). The length of the binding strands was calculated as follows:

$${l}_{{\rm{strand}}}=\sqrt{2{L}_{{\rm{p}}}^{2}\left(\frac{{L}_\mathrm{c}({\rm{ssDNA}})}{{L}_\mathrm{p}}-1+\exp\left({-\frac{{L}_\mathrm{c}({\rm{ssDNA}})}{{L}_\mathrm{p}}}\right)\right)}+\frac{1}{2}{L}_\mathrm{c}({\rm{dsDNA}}).$$

We then employed a geometric monomer model to identify sets of binding-strand lengths that would form a specific cone shape and enable monomers to bind at a defined angle. The 30-nm and 60-nm monomer models were assigned a height of 18 nm and widths of 28.5 nm and 57 nm, respectively. Subsequently, we assigned appropriate binding-strand sequences to each required linker length. Initial naive diameter predictions were refined using container- and tube-specific calibration functions derived from experimental data. Relevant Python scripts are available via our GitHub repository^53^.

### oxDNA simulations

DNA origami structures were exported from scadnano^54^ and prearranged using oxView^55^. The forces used during structure relaxation were also generated in oxView. After a short Monte Carlo pre-relaxation step, we employed molecular dynamics simulations (with the oxDNA2 model^56^) to first relax and then simulate the monomer structure. We generated PDB files for rendering in ChimeraX^57^ with the oxDNA analysis tools command line script^58^. The monomer structure presented in this Article represents a mean structure.

### DNA origami design and folding

We adapted the DNA origami structures developed by Wickham et al.^31^ using scadnano^54^. Structures were folded with a p2873 (30-nm Dipid) scaffold (provided by Prof. Hendrik Dietz’s group; 100 nM in double-distilled H_2_O) or a p7249 (60-nm Dipid) scaffold (Tilibit, 100 nM in 10 mM tris base and 1 mM ethylenediaminetetraacetic acid (EDTA)) and various sets of staple strand oligonucleotides (Integrated DNA Technologies; 200 μM in 20 mM tris and 0.1 mM EDTA, pH 8.0). Folding reactions of 30-nm Dipids were prepared with a final scaffold concentration of 50 nM and a staple strand concentration of 200 nM in FOB18 buffer (5 mM tris, 1 mM EDTA, 18 mM MgCl_2_ and 5 mM NaCl). Folding reactions of 60-nm Dipids were prepared with a scaffold concentration of 34.5 nM and a staple concentration of 277 nM in FOB18. Annotated scadnano files and the scaffold sequences are available via our GitHub repository^53^. Folding solutions were annealed in a thermocycler (Eppendorf, Mastercycler Nexus GX2) using different annealing ramps for different designs. For the structures shown in Figs. 1 and 2 and Extended Data Figs. 4 –6 and 8, the protocol was 15 min at 70 °C followed by a decrease of 0.1 °C every 18 min from 65 °C to 20 °C. The S containers with high disassembly temperatures were folded at 65 °C for 15 min followed by a decrease of 0.1 °C every 15 min from 56 °C to 30 °C. For all other 30-nm Dipid structures, the protocol was 15 min at 60 °C followed by a decrease of 0.1 °C every 6 min from 56 °C to 53 °C. The protocol for the 60-nm Dipid structures was 15 min at 65 °C followed by a decrease of 0.1 °C every 18 min from 60 °C to 40 °C. The gelpy Python package^59^ was used to analyse the preliminary agarose gels and identify suitable folding conditions.

### DNA origami purification

Two purification methods were employed: ultrafiltration and polyethylene glycol (PEG) precipitation. Each approach was used as outlined below.

#### Ultrafiltration purification

Folded samples were purified by ultrafiltration in 32 °C FOB5 washing buffer (5 mM tris, 1 mM EDTA, 5 mM MgCl_2_ and 5 mM NaCl) using Amicon Ultra filters (0.5 ml at 100 K; Millipore). All centrifugation steps were done in a pre-heated centrifuge (Eppendorf, Centrifuge 5425R) at 32 °C. After pre-washing using 500 μl FOB5 buffer, the filters were loaded with 430 μl of FOB5 buffer and 60 μl of folding solution and centrifuged, and the flowthrough was subsequently discarded. This step was repeated with the addition of 450 μl of FOB5 buffer and finally with 450 μl of room-temperature FOB18 assembly buffer. We extracted the purified and buffer-exchanged samples from the filters by repeatedly aspirating and dispensing the solution to dissolve any pellets.

#### PEG precipitation purification

Up to four folding reactions of the same sample were combined, and FOB18 buffer was added up to a volume of 750 μl. This was mixed with 750 μl of precipitation buffer (15% w/v PEG, 500 mM NaCl and ×1 FOB0) and centrifuged at 20,000*g* for 30 min at 25 °C. After centrifugation, the supernatant was removed, and the pellet was resuspended in 750 μl of FOB18 buffer by incubating at 30 °C and 500 rpm for 30 min. Subsequently, another 750 μl of precipitation buffer was added, and the centrifugation step was repeated. The supernatant was discarded, and the sample was resuspended in 60 μl of FOB18 by incubating at 30 °C and 500 rpm for at least 30 min.

### Optional reannealing after purification

After purification and buffer adjustment to FOB18, we reassembled the DNA origami samples in a thermocycler (Eppendorf, Mastercycler Nexus GX2), as planes, tubes and containers can already form during folding. We destabilized the structures by heating them to 38 °C (for structures shown in Figs. 1 and 2 and Extended Data Figs. 4–6 and 8) or disassembled them at 46 °C for 30 min, followed by an annealing ramp of −0.1 °C every 12 min from 38 °C to 20 °C or −0.1 °C every 18 min from 46 °C to 38 °C (28-h protocol) or to 30 °C (48-h protocol, default).

### Negative-stain TEM

For TEM imaging, we incubated 5 μl of DNA origami samples on glow-discharged (coating time 20 s, coating current 35 mA and negative polarity) formvar carbon Cu400 TEM grids (Science Services) for 30–300 s, depending on DNA origami concentrations. We prepared the staining solution by adding 1 μl of 5 M NaOH to 200 μl of 2% uranyl formate solution, followed by vortexing and centrifugation at 5 °C and 21,000*g* for 5 min. After incubating the samples, we washed the grids with 5 μl of stain and then incubated them with 15 μl of stain for 30 s. All negative-stain TEM imaging and tilt-series acquisition was performed using a FEI Tecnai T12 microscope (120 kV) equipped with a Tietz TEMCAM-F416 camera and operated with SerialEM. Tilt series were recorded between ±48° in 2° increments.

### Postprocessing of TEM micrographs

We applied band-pass filter cutoffs with a lower boundary of 1–3 px and an upper boundary of 60–1,000 px. Micrographs were locally contrast-adjusted to improve structure visibility, with or without previous band-pass filtering. For the ‘Enhance Local Contrast’ function in Fiji^60^ (CLAHE), we used block sizes between 32 px and 127 px, a maximum slope of 3 or 4, and histogram bins set to 512. Tomograms were processed with ETOMO (IMOD)^61^. We pre-aligned the tilt series by computing cross-correlations of the image stack and refined the alignment using a fiducial marker model generated by patch-tracking. Tomograms were reconstructed using filtered back-projection with a Gaussian filter cutoff between 0.3 and 0.4 and a fall-off of 0.035.

### Extracting radii from TEM micrographs

We recorded TEM micrographs for each tube and container design. Containers were segmented using a monomer template-matching script, available via our GitHub repository^53^. Briefly, an ellipse was fitted to each segmented container, and the principal diameters of the ellipse were extracted.

Tube diameters were manually measured in Fiji, following two guidelines:

1. Diameters of the same tube were measured with a minimum distance of twice the average tube diameter.
2. Diameters were measured only at positions where the local tube diameter appeared uniform over a width of at least one tube diameter.

The extracted diameters *d*_2D_ were used to calculate the diameters of the assumed 3D ellipsoidal structures *d*_major_ and *d*_minor_ with ${d}_{{\rm{minor}}}={2{d}_{{\rm{2D}}}}/{\uppi }$. Assuming equal minor diameters, we computed *d*_major_ by numerically fitting Ramanujan’s ellipse perimeter formula to the container circumference along the major 2D axis.

### Sample and grid preparation for cryo-ET

DNA origami samples (XS, S and M) were folded, purified and reannealed as described above. Up-concentration was performed using Amicon Ultra filters (0.5 ml and 100 K; Millipore). For several iterations, 60 μl of reannealed DNA origami solution (10 nM) was added to the filter unit, followed by centrifugation at 10,000*g* for 10 min. Further aliquots of the same Dipid solution were added to the same filter unit and centrifuged under the same conditions. The final DNA origami concentrations were *c*_XS_ = 110 nM, *c*_S_ = 440 nM and *c*_M_ = 400 nM. Then, 3 μl DNA origami samples were applied to copper electron-microscopy grids with Quantifoil R 3.5/1 holey carbon film 200-mesh covered with a home-made 3-nm-thick continuous carbon film. Grids were glow-discharged (4 mA for 10 s), blotted and plunge-frozen in liquid ethane using a Vitrobot Mark IV (Thermo Fisher) operated at 95% humidity and 22 °C. In one experiment, eight grids were prepared for XS, S and M samples.

### Cryogenic tilt-series acquisition

Tilt series were acquired using the Tomo5 software on a Krios G4 electron microscope equipped with a cold field emission gun (operated at 300 kV), a Falcon IVi camera and a Selectris X energy filter (Thermo Fisher). Tilt series were acquired at a magnification of ×81,000, corresponding to a pixel size of 1.63 Å. Tilt series were acquired from −60° to +60° in 2° tilt increments using a dose-symmetric tilt scheme. The total dose was 122 e^−^ Å^−^^2^, and the target defocus varied between −2.5 μm and −4 μm. Data were collected in EER format. In total, one grid was imaged for the XS size, two grids for the S size and one grid for the M size. Altogether, 97, 109 and 38 tilt series were collected for the XS, S and M samples, respectively.

### Tomogram reconstruction and processing

Tomograms were reconstructed using RELION-5^62^. Frames were aligned using the implementation of MotionCor2 in RELION^63^. Contrast transfer function (CTF) calculation was performed with CTFFIND4.1^64^. Aligned tilt series were then manually inspected, and bad tilts were removed. The tilt series were aligned using AreTomo^65^ with a pixel size of 10 Å. Templates were matched on these tomograms using PyTom-match-pick^37,38^. The mean atomic structure from an oxDNA simulation was filtered to a resolution of 20 Å and used as a template model. The template-matching results were superimposed on the tomograms and inspected with ChimeraX^57^ using the ArtiaX plug-in^66^. False positive and encapsulated particles were manually removed from the subset of tomograms used to calculate the porosity of the compartments. The coordinates and orientations of the selected Dipids were refined in RELION-5 before analysis.

### Cryo-ET-based pore-size calculations

The custom Python script developed to estimate pore sizes between DNA origami particles in reconstructed tomograms is described in detail in Supplementary Note 2 and is available via our GitHub repository^53^.

### HIV-assembly-inspired simulation

We implemented in Python the bead-spring simulation described by Levandovsky and Zandi^40^. The simulation code is described in Supplementary Note 3 and is available via our GitHub repository^67^.

### Fluorescence microscopy

We imaged DNA origami samples using either a Nikon Ti-2E inverted fluorescence microscope (NIS elements software, SOLA SM II LED light source and Andor NEO 5.5 camera) or, for higher-resolution images, a Leica TCS SP8 laser scanning confocal microscope (LAS X software). Images were globally contrast-adjusted using Fiji. Z-stack projections were also generated using Fiji.

### Coating microscope slides with PDL

We coated slides with poly-D-lysine (PDL) to enhance structure attachment and reduce movement during imaging. Specifically, 50 μl of 0.1 μg ml^−1^ PDL solution was pipetted into microscopy slide wells (Ibidi, Ibidi-treat μ-slide 18-well flat) and incubated for 1 min at room temperature. The PDL solution was removed, and wells were washed twice with 70 μl double-distilled H_2_O before being left to dry at room temperature. Before imaging, wells were rehydrated with 15 μl of ×1 FOB18 buffer. Then, 5 μl of 10 nM DNA origami samples were added to the buffer in the wells, and the chambers were sealed with silicone glue (Picodent, twinsil 22).

### Dipid domain formation in binary mixtures

Folded and PEG-purified Dipid solutions (10 nM, FOB18) were mixed in ratios specified in the respective figures and co-annealed using the default reannealing protocol. For the affinity-driven Dipid domain formation experiments, we recorded *n* confocal images using identical imaging settings (1,024 px × 1,024 px, 200 lps scan speed, two line averages, frame-sequential recording, 488-nm laser at 1.1% power, HyD detector at 100.2% gain, 565-nm laser at 1.6% power, HyD detector at 100.4% gain and ×9 digital zoom) for pure (*n* = 25) and binary (*n* = 100) mixtures of 100% Dipid-A (Atto565), 100% Dipid-B (Atto488), 50% Dipid-A (Atto565) plus 50% Dipid-A (Atto488), and 50% Dipid-A (Atto565) plus 50% Dipid-B (Atto488). We skeletonized the membrane segments and separately averaged the fluorescence intensities in both channels orthogonal to the membrane profile (half-width = 20 px ≈ 250 nm). We used the homogeneously mixed A–A sample to calibrate both channels, assuming an equal number of Atto488- and Atto565-labelled A-Dipids in each pixel. The intensities of the example line profile in Fig. 4b were globally min-max normalized. The purity of the B domain was computed from the calibrated skeleton intensities *I*_c_ as *I*_c,488_/(*I*_c,488_ + *I*_c,565_)

### Porosity tests

After purification and reannealing, 3 μl of XXL Dipid containers in FOB18 were loaded into low-volume channels (Ibidi, μ-Slide VI 0.1 ibiTreat). Next, 1 μl of 10 mg ml^−1^ FITC-labelled dextran solutions (Sigma-Aldrich, 3,000–5,000 g mol^−1^ or 2 × 10^6^ g mol^−1^) were added to one side of a channel to create dextran gradients. Confocal images were recorded at areas with high ambient dextran concentrations. Line profiles from Dipid and dextran channels were extracted with Fiji, individually normalized (${I}_{{\rm{norm}}}={{I}_{{\rm{raw}}}}/{{{{I}}}_{\max }}$) and smoothed with a moving window average.

### In vitro transcription

The two functional modules used in the IVT experiment were derived from the XXL Dipid (Atto655) design. For the IVT module, a staple strand pointing into the container was extended by 20 nt to bind either a partially double-stranded DNA template encoding the dBA or a scrambled version as a negative control. For the membrane-localization module, the same staple strand was extended with a 20-nt capture sequence. After folding and PEG purification, Dipid containers were prepared by reannealing IVT modules (or the scrambled version) and membrane-localization modules together in a 1:9 ratio. Each 20-μl IVT reaction contained 2 μl T7 RNA polymerase (New England Biolabs, 50 Uμl), 2 μl RNA polymerase reaction buffer (New England Biolabs, ×10), 0.4 μl deoxynucleoside triphosphate (dNTP) mix (New England Biolabs, 10 mM per dNTP), 0.5 μl RiboLock RNase inhibitor (Thermo Fisher Scientific, 40 Uμl), 1 μl of 1 M KCl, 2 μl of 400 μM DFHBI-1T dye (Sigma-Aldrich), nuclease-free water, and either 10 μl of 20-nM Dipids or FOB18 as blank. Technical triplicates were transferred into a black 384-well polystyrene plate with a transparent bottom (Brand) and sealed. Notably, low dNTP concentrations were necessary to prevent the disintegration of the containers, which can result from the considerable off-target activity of T7 RNA polymerase^68^. Fluorescence was measured in a BMG CLARIOstar plate reader at 37 °C with an excitation wavelength of *λ*_ex_ = 472 ± 30 nm and and emission wavelength of *λ*_em_ = 520 ± 15 nm. For confocal imaging, reactions were incubated at 37 °C in a thermocycler (Eppendorf, Mastercycler Nexus GX2) for 2 h before imaging (*λ*_ex,dBA_ = 488 nm, *λ*_em,dBA_ = 501–622 nm, *λ*_ex,Atto655_ = 638 nm and *λ*_em,Atto655_ = 649–800 nm) in PDL-coated microscopy slide wells.

### Encapsulation of structures within XXL containers and bilayer formation

Preassembled structures were encapsulated using the XXL-Dipid-derived membrane-localization module (XXL-handle), which includes a ssDNA staple extension pointing into the container. This extension is complementary to matching extensions on the structures to be encapsulated. S-hdT containers were preassembled, PEG-purified and reannealed at a monomer concentration of 10 nM using the standard protocols. Each S-hdT monomer carried the complementary handle extension. Octahedrons were kindly provided by the group of Prof. Dietz and carried 24 extensions per structure, one on each monomer corner. Their assembly followed the procedure in ref. ^14^, in which the triangle interfaces were stabilized by ultraviolet welding^69^. Encapsulation mixtures had a total DNA origami concentration of 10 nM in FOB18 buffer. Component ratios are specified in the corresponding figures. All mixtures were reannealed using the standard protocol.

### Moiré pattern analysis

Fast Fourier transforms were computed in Fiji from unprocessed TEM micrographs using a circular region of interest. We determined the rotational angle of overlapping hexagonal grids in each image based on the innermost maxima of the corresponding fast Fourier transform.

### Large language model tools

We used the online tool ChatGPT^70^ to assist with programming.

## Online content

Any methods, additional references, Nature Portfolio reporting summaries, source data, extended data, supplementary information, acknowledgements, peer review information; details of author contributions and competing interests; and statements of data and code availability are available at 10.1038/s41563-025-02418-0.

## Supplementary information

Supplementary Information Supplementary Note 1–3 and Table 1.

## Data Availability

All relevant data are provided in the Article, the Supplementary Information, our GitHub repository at https://github.com/ckarfusehr/DNA_origami_membranes (ref. ^53^; GH) and figshare at 10.6084/m9.figshare.30339610.v1 (ref. ^71^; FS). The data are also available from the corresponding author upon reasonable request. Specifically: Fig. 1 — DNA sequences and Dipid design files: GH. Fig. 2 — TEM micrographs (panels b and c): FS; extracted size distribution data (panel c): GH. Fig. 3 — ArtiaX files encoding monomer positions (panels a–c): GH; extracted pore-size data: GH; diameter distribution data (panels g, h, j): GH; extracted simulation data (panel j): SI and GH. Fig. 4 — Analysed confocal images (panels b and c): FS; extracted data (panel c): GH; analysed TEM micrographs (panels d, f, g): FS; extracted data (panels d, g): GH. Fig. 5 — IVT plate reader data (panel c): GH.

## Code availability

Python scripts used for DNA-sequence generation, container segmentation, container and tube calibration curve-fitting, cryo-ET pore-size analysis, affinity-driven domain formation analysis, and mechanical property estimation are publicly available via our GitHub repository at https://github.com/ckarfusehr/DNA_origami_membranes (ref. ^53^). The HIV-inspired simulation code is publicly available via a dedicated GitHub repository at https://github.com/ckarfusehr/dipid_shell_simulation (ref. ^67^).
