# Supplementary material for: Self-assembled cell-scale containers made from DNA origami membranes
Source: Nat Mater. 2025 Nov 28;25(3):502–10. doi: 10.1038/s41563-025-02418-0 (PMC12960257; doi:10.1038/s41563-025-02418-0)
Supplement: Supplementary file 1 — Supplementary Note 1–3 and Table 1. [file 41563_2025_2418_MOESM1_ESM.pdf]

nature materials

Supplementary information

https://doi.org/10.1038/s41563-025-02418-0

Self-assembled cell-scale containers made from DNA origami membranes

In the format provided by the authors and unedited

Contents

1 Supplementary Notes **2**

1.1 Supplementary Note 1: Tube formation with controlled mean diameters 2

1.2 Supplementary Note 2: Cryo-ET-based pore size calculation script 3

1.3 Supplementary Note 3: HIV assembly inspired simulation code 3

2 Supplementary Tables **3**

2.1 Supplementary Table 1: HIV inspired simulation statistics 3

1 Supplementary Notes

1.1 Supplementary Note 1: Tube formation with controlled mean diameters

Motivated by our observation that larger Dipid designs (XL, XXL) preferentially form elongated assemblies, we adapted the monomer design to deliberately produce open tubes with controlled diameters. Since tubes can be tiled by a simple hexagonal lattice, we simplified the Dipid interaction profile to use only one of the two six-fold binding axes, reducing the number of binding strands from 30 to 18. We implemented the two principal tube curvatures $\kappa_\perp = r_\mathrm{tube}^{-1}$ and $\kappa_\parallel = 0$ by combining two planar and four conical binding strand subsets with orthogonal sticky domains (Extended Data Fig. 5a). Applying a straightforward geometric correction factor, we could use our list of binding strands, originally designed for closed containers, to generate tubes with 62 unique target tube diameters $d_\mathrm{tube} = d_\mathrm{container} \cos(30°)$ (Extended Data Fig. 5a). As for the containers, we chose six Dipid designs at random (XS, S, M, L, XL, XXL) and analyzed the resulting assemblies using negative stain TEM and confocal microscopy (Extended Data Fig. 5b-e). We also observed the expected increase in polydispersity with increasing tube diameters (Fig. 3g) [1, 2]. In the initial growth phase, the Dipids assemble into a curved sheet that statistically closes into a tube seed at one point. The polydispersity of the larger tube sizes is the result of the larger fluctuations of the initial sheets. Due to these fluctuations, a curved sheet can close back on itself to form a tube before, during, or after accumulating the 'correct' number of monomers for its target diameter. Notably, tubes may form with different chiralities, depending on whether the tube is closed precisely along the direction defined by the curvature of the monomers or with a slight shift. Under the TEM, we consistently observed achiral tubes as well as left-handed tubes with varying degrees of shift (Extended Data Fig. 5f and 6) and associated Moiré patterns. We also encountered other growth irregularities such as branching, bending, and the growth of structures from defects, potentially leading to multi-layer tubes (Extended Data Fig. 5g). Aggregation or interconnection of several tubes might result from the incorporation of occasionally occurring DNA origami dimers or due to salt-mediated electrostatic attraction between tubes (Extended Data Fig. 5g). If tight geometry control is required for a specific application, the polydispersity and defect frequency of tubular assemblies might be reduced by increasing the number of distinct monomers, at the cost of assembly time [3, 4]. Unlike previously reported tube-forming DNA origami assembled via end-to-end stacking of reconfigurable rings, which offer virtually no variation in diameter [5], or hexagonal tile-based assemblies that yield only seven distinct diameters between approximately $166\,\mathrm{nm}$ and $710\,\mathrm{nm}$ after correction to three-dimensional geometry [6], our lateral, side-to-side assembly strategy uniquely enables formation of tubes spanning a broader diameter range from $88\,\mathrm{nm}$ to $767\,\mathrm{nm}$ and provides considerably finer resolution with more than 60 predicted intermediate diameters.

1.2 Supplementary Note 2: Cryo-ET-based pore size calculation script

The custom Python script was developed using several open-source libraries, including PyVista for 3D mesh manipulation and visualization, Shapely for 2D geometric operations, SciPy for numerical analysis, and NetworkX for graph-based neighborhood analysis. The script extracts isosurfaces from MRC volumes using a marching cubes algorithm and applies 3D transformations to position each mesh according to the particle coordinates and Euler angles parsed from the RELION STAR files. The script accepts STAR particle files after picking or template matching, as well as STAR files after refinement as input. For each group of particles, the local 3D configuration is projected onto a best-fit plane to create a 2D polygonal representation of the interparticle space. Pentagons and hexagons are automatically detected by the script. It also allows the creation of user-defined custom particle groups in the form of an array with the list of desired particles. The pore size was then estimated by calculating the area of the largest inscribed circle within this gap polygon. In the case of pentagons, there is only one inscribed circle. In the case of hexagons with a central particle, there are 6 inscribed circles formed by each trimer of particles. For custom groups, the script differentiates between trimers or subgroups with more particles to best adjust the size of the inscribed circles. For each automatically detected or specified polygon, the script generates a 2D representation of the polygon, a 3D view of the scene with the polygon and only the particles involved, as well as their neighbor at rank N+1. For each coordinate file, a CSV file is generated with all the surfaces, distances, and dimensions of each particle group and polygon.

1.3 Supplementary Note 3: HIV assembly inspired simulation code

We rescaled the dimensions of the original triangular prisms to represent the center-to-center distances in Dipid trimers, which depend on the specific cone angles. The main simulation loop first checks whether the container has closed. If the container remains open, the code assesses for closure events (e.g., pentagon closures). If none are detected, then in 95% of cases, a new prism is added at the boundary position with the smallest opening angle between monomers. In the remaining 5%, the prism is added at a random boundary position. The system is equilibrated by performing batches of Langevin simulation steps without noise. We evaluated equilibration by measuring the energy drift per node between batches; once the drift dropped below a preset relative threshold, the system was considered equilibrated. Finally, the simulation ensures valid network connectivity. This process repeats until the container is closed or the maximum number of simulation steps is reached.

2 Supplementary Tables

2.1 Supplementary Table 1: HIV inspired simulation statistics

Table 1: Overview of simulation runs per α_{sim}. These data, as well as the per-simulation data, are available as CSV files on our GitHub repository

alpha sim	random placement prob	#sims	#sims container	#sims non-closed object	mean aspect ratio container	median aspect ratio container	std aspect ratio container
4.0	0.05	4.0	0.0	4.0			
4.3	0.05	4.0	0.0	4.0			
4.4	0.05	4.0	0.0	4.0			
4.6	0.05	4.0	0.0	4.0			
4.8	0.05	4.0	0.0	4.0			
5.0	0.05	4.0	0.0	4.0			
5.2	0.05	4.0	0.0	4.0			
5.4	0.05	4.0	0.0	4.0			
5.6	0.05	4.0	0.0	4.0			
5.8	0.05	4.0	0.0	4.0			
6.0	0.05	14.0	0.0	14.0			
6.1	0.05	38.0	0.0	38.0			
6.2	0.05	39.0	0.0	39.0			
6.3	0.05	51.0	1.0	50.0	3.53	3.53	
6.4	0.05	48.0	3.0	45.0	2.18	2.26	0.23
6.6	0.05	50.0	21.0	29.0	1.92	1.88	0.41
6.8	0.05	87.0	43.0	44.0	1.72	1.75	0.32
7.0	0.05	54.0	36.0	18.0	1.48	1.48	0.25
7.2	0.05	54.0	42.0	12.0	1.61	1.5	0.79
7.4	0.05	54.0	43.0	11.0	1.36	1.27	0.27
7.6	0.05	57.0	53.0	4.0	1.29	1.22	0.22
7.8	0.05	59.0	48.0	11.0	1.25	1.2	0.17
8.0	0.05	78.0	70.0	8.0	1.25	1.19	0.17
8.5	0.05	48.0	48.0	0.0	1.13	1.12	0.09
9.0	0.05	30.0	30.0	0.0	1.1	1.09	0.06
10.0	0.05	30.0	30.0	0.0	1.11	1.08	0.09
11.0	0.05	30.0	29.0	1.0	1.1	1.09	0.04
12.0	0.05	30.0	30.0	0.0	1.11	1.1	0.07
13.0	0.05	30.0	28.0	2.0	1.09	1.08	0.06
14.0	0.05	30.0	29.0	1.0	1.1	1.09	0.05
15.0	0.05	40.0	40.0	0.0	1.1	1.09	0.06
16.0	0.05	30.0	30.0	0.0	1.09	1.08	0.07

alpha sim	random placement prob	#sims	#sims container	#sims non-closed object	mean aspect ratio container	median aspect ratio container	std aspect ratio container
17.0	0.05	30.0	30.0	0.0	1.08	1.09	0.05
18.0	0.05	30.0	30.0	0.0	1.11	1.12	0.05
19.0	0.05	30.0	30.0	0.0	1.07	1.06	0.03
20.0	0.05	30.0	30.0	0.0	1.08	1.08	0.03
21.0	0.05	20.0	20.0	0.0	1.07	1.07	0.04
22.0	0.05	20.0	20.0	0.0	1.1	1.09	0.04
23.0	0.05	20.0	20.0	0.0	1.06	1.03	0.06
24.0	0.05	20.0	20.0	0.0	1.11	1.1	0.04
25.0	0.05	19.0	19.0	0.0	1.1	1.09	0.04
26.0	0.05	20.0	20.0	0.0	1.15	1.17	0.07
27.0	0.05	20.0	20.0	0.0	1.13	1.15	0.05
28.0	0.05	20.0	20.0	0.0	1.13	1.11	0.06
29.0	0.05	20.0	20.0	0.0	1.11	1.09	0.08
30.0	0.05	20.0	20.0	0.0	1.08	1.07	0.06
31.0	0.05	20.0	20.0	0.0	1.1	1.07	0.06
32.0	0.05	20.0	20.0	0.0	1.08	1.06	0.04
33.0	0.05	20.0	20.0	0.0	1.07	1.06	0.04
34.0	0.05	20.0	20.0	0.0	1.07	1.05	0.03
35.0	0.05	20.0	20.0	0.0	1.07	1.05	0.04

References

[1] Helfrich, W. Size distributions of vesicles : the role of the effective rigidity of membranes. *Journal de Physique* **47**, 321–329 (1986). URL http://dx.doi.org/10.1051/jphys:01986004702032100.

[2] Levandovsky, A. & Zandi, R. Nonequilibirum Assembly, Retroviruses, and Conical Structures. *Physical Review Letters* **102**, 198102 (2009). URL https://link.aps.org/doi/10.1103/PhysRevLett.102.198102.

[3] Videbæk, T. E. *et al.* Tiling a tubule: how increasing complexity improves the yield of self-limited assembly. *Journal of Physics: Condensed Matter* **34**, 134003 (2022). URL https://dx.doi.org/10.1088/1361-648X/ac47dd.

[4] Videbæk, T. E. *et al.* Economical routes to size-specific assembly of self-closing structures. *Science Advances* **10**, eado5979 (2024). URL https://www.science.org/doi/10.1126/sciadv.ado5979.

[5] Kucinic, A., Huang, C.-M., Wang, J., Su, H.-J. & Castro, C. E. DNA origami tubes with reconfigurable cross-sections. *Nanoscale* **15**, 562–572 (2023). URL https://doi.org/10.1039/D2NR05416G.

[6] Wang, P. *et al.* Programming Self-Assembly of DNA Origami Honeycomb Two-Dimensional Lattices and Plasmonic Metamaterials. *Journal of the American Chemical Society* **138**, 7733–7740 (2016). URL https://doi.org/10.1021/jacs.6b03966.
